# Efficacy of the Unani Regimen as an Add-On to Standard Treatment in Hospitalised RT-PCR-Confirmed Mild to Moderate COVID-19 Patients: An Open-Label Randomized Controlled Trial

**DOI:** 10.7759/cureus.38574

**Published:** 2023-05-05

**Authors:** Jugal Kishore, Rohit Kumar, Tamanna Nazli, Aftab Ahmad, Pawan Kumar, Asim A Khan

**Affiliations:** 1 Department of Community Medicine, Vardhman Mahavir Medical College and Safdarjung Hospital, New Delhi, IND; 2 Department of Pulmonary Critical Care and Sleep Medicine, Vardhman Mahavir Medical College and Safdarjung Hospital, New Delhi, IND; 3 Department of Unani Medicine, Vardhman Mahavir Medical College and Safdarjung Hospital, New Delhi, IND; 4 Department of Pathology, Central Council for Research in Unani Medicine, New Delhi, IND; 5 Department of Unani Medicine, Central Council for Research in Unani Medicine, New Delhi, IND

**Keywords:** temperament, whoqol-bref, national early warning score (news), ayush, immunomodulatory, unani, covid-19, rt-pcr, unani joshanda, tiryaq-e-arba

## Abstract

Background

The coronavirus disease 2019 (COVID-19) pandemic resulted in mortality and morbidity worldwide. Many treatment modalities have been experimented with limited success. Therefore, the traditional system of medicine needs to be explored.

Objective

To evaluate the benefits of Unani regimens*Tiryaq-e-Arba* and Unani Joshanda, as adjuvant therapy, were compared to standard treatment alone among reverse transcription polymerase chain reaction (RT-PCR)-confirmed mild to moderate COVID-19 cases.

Materials and methods

An open-label, double-arm, randomized, controlled interventional clinical study was conducted among 90 RT-PCR-confirmed mild to moderate COVID-19 inpatients admitted to a tertiary care hospital in New Delhi, India. Participants who fulfilled the criteria for inclusion were randomly assigned to two arms, with 43 subjects allocated to the Unani add-on arm and 47 subjects to the control arm receiving standard treatment alone.

Results

Clinical recovery was achieved in all patients of the Unani arm, while in the control arm, three (6.4%) patients deteriorated and had to be shifted to ICU following admission. In the intervention arm, a shorter duration of hospitalization was observed (mean 5.95 days {SD = 1.99}) than in the control arm (mean 7.62 days {SD, 4.06}); which was a statistically significant difference (p-value 0.017). The majority of the patients recovered within 10 days in the Unani add-on arm. The number of days taken for the reduction of symptoms was significantly less in the intervention arm (mean 5.14 days {SD, 2.39}) as compared with standard treatment (mean 6.53 days {SD, 3.06}) (p < 0.02). Renal and liver safety parameters were within the normal limits in both arms and no serious adverse event was reported.

Conclusion

Adding Unani formulations to standard treatment significantly reduced the duration of hospital stay and showed early recovery in COVID-19 patients compared with the control arm. It may be concluded that the synergistic effect of the Unani add-on with standard treatment gave more promising results in mild to moderate COVID-19 patients.

## Introduction

Coronavirus disease 2019 (COVID-19), caused by a novel coronavirus, Severe Acute Respiratory Syndrome Coronavirus 2 (SARS-CoV-2), resulted in a catastrophe in recent times. It has been estimated to have infected more than 678 million people, with around 6.7 million deaths globally as on 16 February 2023 [1].

During the course of the pandemic, several treatment modalities have been evaluated for COVID-19, which include the use of remdesivir, ivermectin, hydroxychloroquine, chloroquine, corticosteroids, lopinavir/ritonavir, favipiravir and convalescent plasma therapy and many more. However, no decisive scientific consensus has been reached regarding the efficacy of many of these drugs in the treatment of COVID-19 in hospitalized and non-hospitalized patients [2]. Later, various types of vaccines were rolled out globally against SARS-CoV-2 infection. These vaccines induce considerable humoral and cellular-mediated immune responses with good efficacy. SARS-CoV-2 is constantly evolving through random mutations that often increase the virus’ ability to evade adaptive immune response leading to an increased risk of reinfection or decreased efficacy of vaccines and the possibility of breakthrough infection even after complete vaccination [3].

Evidence from available data reveals that the clinical manifestations of COVID-19 infection include flu-like symptoms, such as fever, headache, myalgia, vomiting, and nausea. These are similar to the symptoms experienced during the ‘epidemic influenza’ (*Nazla-e-Wabāiya*). The Unani preparations have been used during epidemics (*Waba*) to treat various infectious diseases (*Amraz-e-Wabaiya*), such as plague, smallpox, and cholera [4]. Unani classical literature such as Alqanoon Fit Tib by Ibne Sina, Kitab-Al-Taisir by Ibne Zohr and treaties of Buqrat Epidemic I, Epidemic III and Airs, Waters and Places lists various drugs for the prevention of diseases through immunomodulation. These preparations have been used during epidemics either singly or more often in combination. The study drug *Tiryaq-e-Arba*, a prophylactic medicine recommended for use before and during epidemics, is one such drug with *dafae taffun *(anti-infective), antidote, and expectorant actions. It is a combination of four herbs comprising *Laurus nobilis* L. (*Habbul ghar*), *Gentiana lutea *(*Juntiyana romi*), *Commiphora myrrha* (*Murr maki*), and *Aristolochia longa *(*Zarawand taweel*) [5-10]. *Tiryaq-e-Arba *along with Unani *Joshanda *(a decoction made with three herbs *Zizyphus jujube, Cordia myxa, *and *Cydonia oblonga*) is advocated during the pandemic by the advisory of the Government of India, released by the Ministry of AYUSH for COVID-19 [10].

All the individual ingredients of the combination exhibit anti-viral activity in addition to anti-inflammatory, antioxidant, and immunomodulatory properties [4-17]. Recently, it was also reported that it exhibits antiviral activity against SARS-CoV-2 [5]. Therefore, *Tiryaq-e-Arba* can be a potential add-on drug for COVID-19 management.

This study was planned to assess the effectiveness and safety of *Tiryaq-e-Arba* plus Unani *Joshanda *as an add-on therapy to the standard treatment in mild to moderate COVID-19 patients as compared to standard management in combination with symptomatic treatment, including anti-viral, as prescribed. The outcome of this study will help the policymakers in AYUSH and Health Ministries to advise the alternative herbal preparation for use in public for mild and moderate COVID-19 infection.

## Materials and methods

Study design and participants

This open-label clinical study was conducted at a dedicated COVID-19 facility of a tertiary care hospital in New Delhi, India after obtaining approval from the Institutional/Central Ethics Committees of the participating site at Safdarjung Hospital, New Delhi, and Central Council for Research in Unani Medicine (IEC/VMMC/SJH/Project/2020-10/CC-75). The trial has been prospectively registered in the Clinical Trial Registry of India (ICMR- NIMS): Reference no. CTRI/2020/12/029575 [18]. All research procedures were strictly adhered to, based on AYUSH Good Clinical Practice (GCP) and Indian Council for Medical Research (ICMR) Guidelines.

Patient enrollment and selection criteria

A total of 343 COVID-19 patients were screened during the trial period. Ninety eligible participants of either sex, aged between 18-65 years, with a confirmed diagnosis of COVID-19 by RT-PCR (in a pharyngeal/nasopharyngeal swab sample), with mild or moderate severity that required hospitalization as decided by the attending physicians were included after obtaining informed written consent. Patients suspected but not confirmed by RT-PCR for SARS-CoV-2, those diagnosed as severe or critical, those with severe primary respiratory disease or pneumonia, and patients having high-impact comorbidities such as cancer, heart disease, stroke, psychiatric disorders, etc. Pregnant and lactating women or those participating in another COVID-19 trial were excluded from the study. Each patient eligible for the study was informed about the nature and purpose of the study prior to recruitment in the study, and after obtaining verbal confirmation to participate, patients were provided a physical informed consent form. The patient (or a caregiver/ hospital attendant in the room) took a photograph of the signed informed consent document and electronically sent it to the investigator.

Randomization and allocation concealment

Once the patient had given written consent to participate in the study, they were randomly allocated to one of the two study arms, viz. Unani add-on arm and the standard treatment arm by a statistician who was not involved in the study. To ensure random allocation, block randomization was used, with permuted blocks of variable block sizes of four, six, and eight. A random number sequence was used to determine the subsequent block permutation for allocation. Patient allocation was concealed, using opaque sealed envelopes. These envelopes, containing the participant allocation, contained a unique key that determined which arm the patient was to be allocated. This process was supervised by the Department of Community Medicine of the hospital at the study site. This being an open-label study, neither the investigators nor the patients were blinded. No placebo was used in the control arm.

Study interventions

Unani add-on (Intervention arm): Patients were given Unani add-on regimen (‘*Tiryaq-e-Arba*’ plus ‘Unani *Joshanda*’) along with the standard allopathic treatment. Unani add-on regimen includes a semisolid Unani pharmacopeial preparation- ‘*Tiryaq-e-Arba*’, an Unani antidote for epidemics which comprises equal proportions of four herbs prepared as per the Unani Formulary, along with ‘Unani *Joshanda*’, a decoction made with three herbs. In order to obtain decoction, all three ingredients of ‘Unani *Joshanda*’, were boiled in 250 ml of water and reduced to one-half (125ml), followed by filtration [4,8,11,15,19,20]. Both the herbal Unani drug preparation were supplied by the Indian Medicines Pharmaceutical Corporation Limited (IMPCL) which is engaged in the Manufacturing and Marketing of Ayurvedic and Unani Medicines and is under the administrative control of the Ministry of AYUSH, Govternment of India. This was given as an add-on regimen to the standard treatment for COVID-19 patients for 14 days, or till discharge from the hospital, whichever was earlier. These drugs were administered in a hospital setting under the supervision of clinical investigators.

Standard treatment (active control arm): The patients were given standard treatment in accordance with the recommended treatment regimens for the novel Coronavirus infection, included in the extant interim Guidelines approved by the Ministry of Health and Family Welfare (MoHFW), Government of India [21]. It includes all or some of the drugs, depending upon the clinical condition of each patient, at the discretion of the treating physician: acetaminophen 500mg, Vitamin C 500mg twice/ day, Zinc 75-125 mg/day, Vitamin D3 6000IU/day, azithromycin 500mg/day for five days, antiviral drug- remdesivir 800 mg or favipiravir 1800mg, Oxygen therapy, in addition to corticosteroids (like dexamethasone 8 mg once to thrice per day or methylprednisolone 20/40mg twice per day), if required. If the patient needed oxygen therapy over the course of the study, oxygen was administered via nasal prongs, a venture mask, or a non-rebreather mask (NRBM). Continuous Positive Airway Pressure (C-PAP) or Bi-PAP was also used when indicated. Patients with multiple organ failure, high oxygen requirement, and those requiring invasive mechanical ventilation were transferred to the intensive care unit (ICU) for further management and discontinued from the study.

Baseline assessment and follow-up

The demographic characteristics of participants, like age, sex, comorbidities, the onset of symptoms, and disease severity, were recorded at baseline during enrolment. A detailed history and physical examination, including temperament (*Mizaj*) assessment, Quality of Life assessment, and baseline investigations were assessed in all patients. Clinical findings, including heart rate, blood pressure, respiratory rate, oxygen saturation, and level of consciousness, along with the assessment of symptoms, were recorded every day during their stay in the hospital. Patients with moderate COVID-19 and those with respiratory distress were continuously monitored until their oxygen saturation levels were within the safe limit. During the hospital stay, daily symptoms were recorded and evaluated using the National Early Warning Signs (NEWS) score to detect any clinical deterioration. Adverse reactions, if any, were recorded and treated conservatively [22]. The patients were discharged from the hospital if they tested negative for COVID-19 using RT-PCR or if they met the revised discharge criteria of being: 1. Afebrile for three consecutive days, 2. Maintaining oxygen saturation above 95% without oxygen support for four days, and 3. 10 days from the onset of symptoms [23]. RT-PCR was repeated on recovery before the time of discharge, usually 10 days in most cases, or earlier tests were sought if recovered before 10 days.

Outcome measures

The study institute does not have provisions to assess the viral load. Therefore, the primary outcome included the duration (time to clinical recovery) of the primary symptoms of COVID-19, including fever, cough, and fatigue. Secondary outcomes included time to negative conversion of RT-PCR test for COVID-19 from randomization, aggravation rate based on National Early Warning Score (NEWS) score, defined as the change in the category of the disease severity and/or requirement of being transferred to the ICU as well as length of hospitalization and duration of ICU stay. The secondary outcome also included the duration of the requirement of supplemental oxygen or any clinical adverse events and change in Quality-of-life scores validated by WHO Quality of Life (Qol)-BREF (WHOQOL-Bref) and temperament (Mizaj) score using a standard questionnaire.

The WHOQOL-Bref is a shorter version of the WHOQOL-100. It is a reliable validated self-administered questionnaire comprising 26 questions on the individual's perceptions of their health and well-being. Responses to questions are on a 1-5 Likert scale where 1 denotes "disagree" or "not at all" and 5 denotes "completely agree" or "extremely". There are also two separate questions that ask specifically about the individual's overall perception of their health and the individual's overall perception of their quality of life [24].

Temperament (*Mizaj*) assessment is an integral concept of Unani medicine. It signifies the metabolic constitution, psychological makeup, and behavioral pattern of an individual. Each person is considered to have a specific humoral makeup, determined by the predominance of “humor” in the body. Based on the dominance of any of the humors, the temperament of a person is determined. When these humors are present in the right proportion in the body, the body remains healthy and any disbalance in quality and quantity of the humor may result in ailment or disease. The temperament (*Mizaj*) of each subject was identified at baseline and before discharge.

*Mizaj a*ssessment was done using a standard questionnaire with 10 items based on the characteristics of a person in relation to skin color, touch, physique (muscle and fat mass), hair color condition, physical functions, activity, diet, sleep, and wakefulness, and lastly, the psychic functions, wherein they were divided into four categories; hot and moist, hot and dry, cold and moist, and cold and dry groups based on their *mizaj *which was predetermined by a team of expert Unani practitioners. The Mizaj has been discussed in detail in the Principles of Unani Medicine by Hakim Ahemed Husain as early as the 1940s [25].

Clinical evaluations

NEWS is a specially designed physiological parameter-based summary score of six ‘vital signs' (respiratory rate, oxygen saturation, systolic blood pressure, heart rate, level of consciousness, temperature) and supplemental oxygen dependency, which is used to identify those at risk of early clinical deterioration in the hospital admitted COVID-19 patients. The use of this score helped to reduce any bias in ICU transfers and allowed the clinician to serially assess the patients in both arms and transfer them to the ICU as a protocol if a deterioration was detected.

Clinical assessment for symptoms like fever (≤36.6°C or axilla, ≤37.2°C oral), breathlessness (respiratory rate ≤24/minute on room air, oxygen saturation (SpO2) >94% on room air), and cough - (on a patient-reported scale). Laboratory assessments, including absolute lymphocyte count (ALC), neutrophil-lymphocyte ratio (NLR), prothrombin time, international normalized ratio (INR), liver function test (LFT), renal function tests (RFT), serum electrolytes, and RT-PCR, were done at admission, discharge, and whenever required.

Sample size

The sample size calculation was based on a study done by Chen et al., in which it was observed that 88% effectiveness of Herbal Medicine combined with Allopathic medicine as compared to antiviral drugs alone, which had 75% effectiveness [26]. Unani preparation is usually prepared from herbs, therefore, taking these values as a reference; the minimum required sample size with 80% power of the study, and one-tailed alpha, is 48 patients in each study arm. The sample is calculated at 48 with an effect size of 13% and 80% power and 5% margin of error, 10% attrition rate is added i.e. 53; however, finally 43 in the intervention arm and 47 in the control arm were enrolled. It was due to a sudden fall in hospital admission, instead of 96 patients, we could finally enroll and follow up 90 patients during the study period. The difference in the number of intervention and control arms was due to block randomization, where the blocks were of varied sizes according to age and gender.

Statistical analysis

The statistical analysis was carried out by SPSS Version 21 (IBM Corp., Armonk, NY). The participants, once enrolled, were analyzed with the intention-to-treat analysis. Baseline characteristics were compared across the two arms using the Chi-square test for categorical measures and Student’s t-test for continuous measures. The primary outcome for days to clinical recovery was compared across the two arms using the Mann-Whitney U test. The secondary outcomes were compared across the two arms using paired t-test for parametric measures and NEWS scores and a Chi-square test or Fisher’s exact test for the categorical outcomes. Mann-Whitney U test was used for continuous outcomes which were non-parametrically distributed. Wherever possible, the outcome measures were compared using a box-and-whisker plot and line charts across the two arms.

## Results

**Figure 1:**
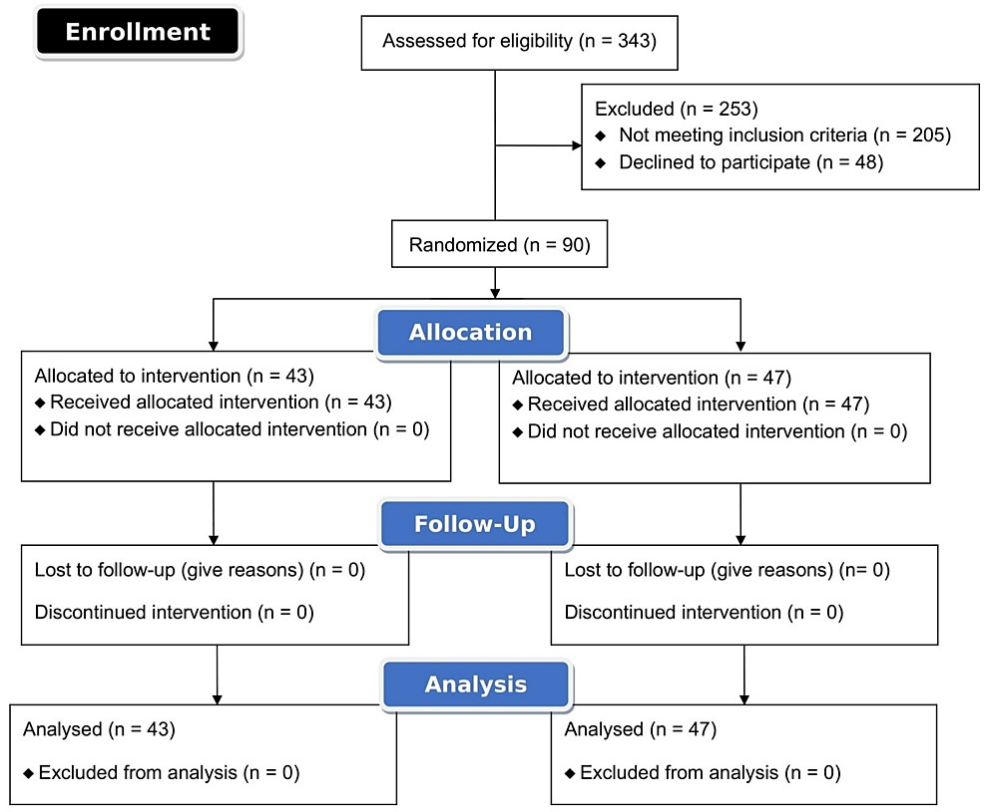
Consort flow chart of recruitment and allocation of patients to Unani and Control arm

During the study period, 343 patients with mild to moderate COVID-19 were admitted and screened for eligibility. Ninety of these patients were enrolled in the study after taking written informed consent. Of the total patients enrolled, 43 were randomized in the Unani arm and 47 were allocated to the control arm, the flow diagram for which is shown in (Figure 1).

None of the patients dropped out of the study in the intervention (Unani add-on) arm. However, three patients in the control arm (6.4%) had to be shifted to ICU in the control arm and were discontinued from the study. The demographic and baseline characteristics were comparable across both groups. The details are given in Table 1.

**Table 1:** Demographic and Baseline characteristics between the study and control arm Data expressed in mean (SD); *data expressed as number (percentage) COVID-19: Coronavirus disease 2019

		Study group (Intervention vs Control)	
		Study arm (Unani add-on) (N = 43)	Control arm (standard therapy) (N = 47)
Age (Mean ±SD)		39.7±12.7	39.5±12.4
Age (range)*			
18-30		12 (27.9)	15 (31.9)
31-40		13 (30.2)	10 (21.3)
41-50		08 (18.6)	11 (23.4)
51-60		09 (20.9)	10 (21.3)
61 and above		01 (2.3)	01 (2.1)
Gender*			
Male		28 (65.1)	29 (61.7)
Female		15 (34.9)	18 (38.3)
Healthcare Workers*		9 (20.6)	15 (31.9)
Comorbidities*			
Diabetes	8 (18.6)		7 (14.9)
Hypertensive	7 (16.3)		10 (21.3)
Others	10 (23.3)		8 (17.0)
Clinical characteristics (Mean ±SD)			
Baseline SpO2 (%)	96.9±1.9		97.0±1.9
Temperature	99.8±2.0		100.6±1.9
Respiratory rate (breaths/min)	19.6±2.1		19.4±1.6
Clinical symptoms*			
Fever	35 (81.4)		45 (95.7)
Dry Cough	28 (65.1)		32 (68.0)
Sore throat	10 (23.8)		9 (19.2)
Fatigue/Malaise	11 (25.6)		15 (31.9)
Body ache	8 (18.6)		17 (36.2)
Headache	4 (9.3)		10 (21.3)
Rhinorrhoea	8 (18.6)		6 (12.8)
Shortness of Breath	21 (48.8)		12 (25.5)
COVID-19 Disease Severity Category (WHO)*			
Mild	32 (74.4)		34 (72.3)
Moderate	11 (25.6)		13 (27.7)

The mean ± SD age of patients was 39.7±12.7 years in the intervention (Unani add-on) arm and 39.5±12.4 years in the control arm; 28 patients (61.1 %) in the Unani arm and 29 (61.7%) in the control arm were men, and this difference was not statistically significant (p = 0.74). The distribution of the total number of study participants is as follows: 24 (26.7%) patients were among Health Care Workers (HCW), 12 (13.3%) patients gave a history of contact with a diseased person positive for COVID-19, and there were only four (4.4%) patients who gave a history of travel in the past month. The most frequent comorbidities observed were hypertension (18.9%) and diabetes (16.7); 66 patients (77.3%) had mild severity, and 24 patients had moderate severity (26.7 %). The most common signs and symptoms at presentation were fever (88.9%), dry cough (66.7%), rhinorrhea (33.3%), and fatigue (28.9%). Baseline laboratory findings were balanced across the two arms. Chest X-Ray (CXR) findings were available for all patients. None of the patients had abnormal CXR findings.

Time to resolution of symptoms

The primary outcome measure was clinical recovery or duration of subsidence of main symptoms of COVID-19 (including fever, cough, and fatigue) from the day of admission or enrolment. The mean duration of symptoms was significantly less in patients who received the course of Unani add-on treatment compared with patients who received only standard treatment in the control arm (Table 2).

**Table 2:** Primary and Secondary Outcomes Data expressed in Mean (SD); p-values were determined using the Mann-Wintney test *data expressed as number (percentage); † Azithromycin 500mg, Augmentin 625mg; ‡ Methyl prednisolone and Dexamethasone (Oral or i.v.); § AEs Adverse effects were not mutually exclusive

Study Outcome variables	Study arm (Unani add-on) (N = 43)	Control arm (Standard therapy) (N = 47)	p-value
Primary outcome			
Patients Clinically recovered*	43 (100)	44 (93.6)	0.34
Secondary outcome			
Clinical Recovery (days)	5.1± 2.4	6.5± 3.1	0.02
Hospital stay (days)	5.9±1.9	7.62±4.1	0.017
Patients discharged before day 10*	41 (95.4)	36 (76.6)	0.011
Patients transferred to ICU*	0 (0)	3 (6.4)	0.24
Duration of O2 Requirement (days)	2.3±1.8	6.0±3.6	0.006
Duration of Prone position (days)	3.3 ±1.5	2.8±1.9	0.67
Treatment received*			
Favipiravir	7 (16.3)	1(2.1)	0.03
Remdesivir	16 (37.2)	16 (34.0)	0.754
Antibiotics †	12 (27.9)	17 (36.2)	0.164
Steroids ‡	18 (41.9)	20 (42.6)	0.387
Supplemental oxygen	12 (27.9)	8 (17.1)	0.215
Prone positioning	3 (6.9)	5 (10.6)	0.348
Clinical AEs during hospital stay*			
Abdominal pain (n = 4)	2 (4.7)	2 (4.3)	0.927
Nausea (n = 4)	1 (2.3)	3 (6.4)	0.351
Malaise (n = 3)	0 (0)	3(6.4)	0.092
Headache (n = 3)	1 (2.3)	2 (4.3)	0.610
Heartburn (n = 1)	0 (0)	1 (2.1)	0.336
Anorexia (n = 2)	1 (2.3)	1 (2.1)	0.949
Total adverse effects §	4 (9.3)	8 (17.0)	0.36

**Figure 2:**
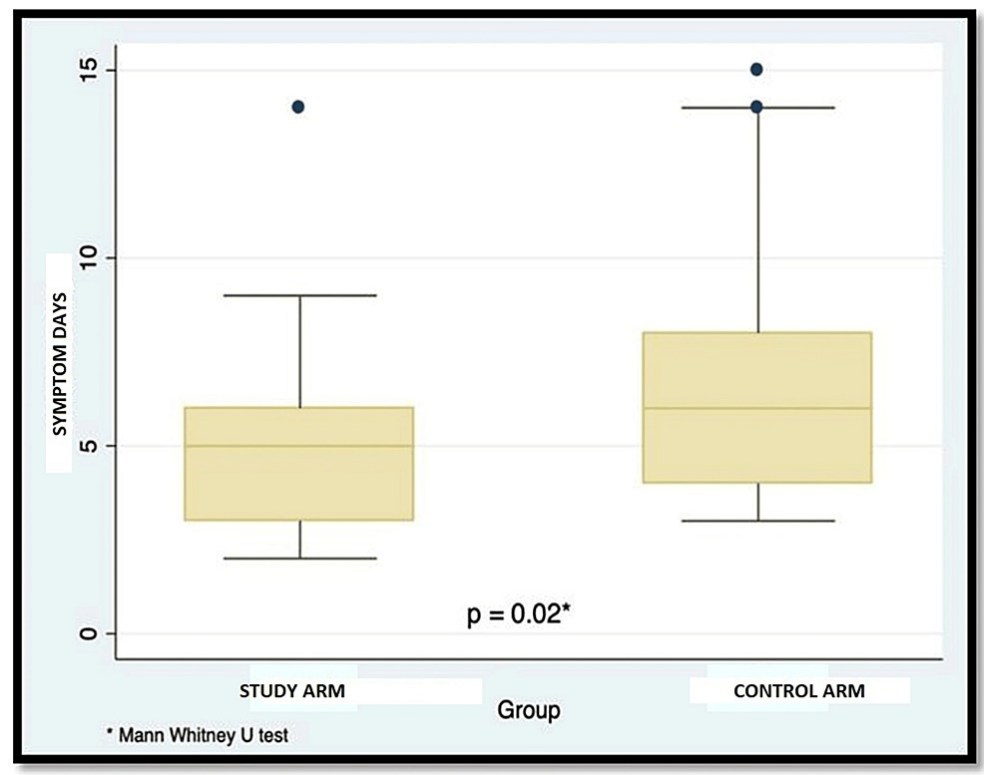
Box plot showing median days of symptoms between the Interventional arms (Unani study arm, and Control arm)

The mean time of resolution of symptoms was 5.14± 2.4 days in the intervention (Unani add-on) arm and 6.53± 3.1 days in the control arm, and this difference was statistically significant (Mann Whitney U test, p = 0.02) (Figure 2).

Time to negative RT-PCR

The mean number of days to negative RT-PCR in the Unani arm was 5.9 ±2.0 and 7.6 ±4.0 in the control arm and the difference between the two was statistically significant (p = 0.01). In the Unani arm, 41 (95%) patients turned RT-PCR negative within 10 days after admission, and only two patients from the Unani arm took more than 10 days to turn negative in the RT-PCR test. In contrast, 36 (76.6%) patients in the control arm turned negative within 10 days and 11 patients took more than 10 days to turn negative. The maximum number of days required to turn RT-PCR negative was 18 days in the control arm, whereas it took 11 days in the intervention (Unani add-on) arm (Table 2).

Rate of conversion to severe cases

In the intervention (Unani add-on) arm, all 43 patients (100%) recovered from COVID-19 disease and none of the enrolled patients progressed to the next stage with regard to severity during the hospital stay. However, as a result of clinical deterioration and a steady rise in the NEWS score, three patients in the control arm (6.4%) were transferred to ICU following admission. However, no deaths were reported in any of the study arms (Table 2).

Length of hospitalization

Patients in the control arm had a significantly longer hospital stay (mean ± SD = 7.6± 4.1) as compared to the Unani arm (mean±SD = 5.9±1.9), and the difference was statistically significant (Mann Whitney U test, p = 0.017). Those in the Unani arm who received the Unani add-on treatment along with the standard treatment stayed for a shorter duration in comparison to the control arm. Consequently, when the maximum length of hospital stay was compared, it was 11 days in the Unani arm in contrast to 18 days in the control arm (Table 2).

National early warning score (NEWS)

**Figure 3:**
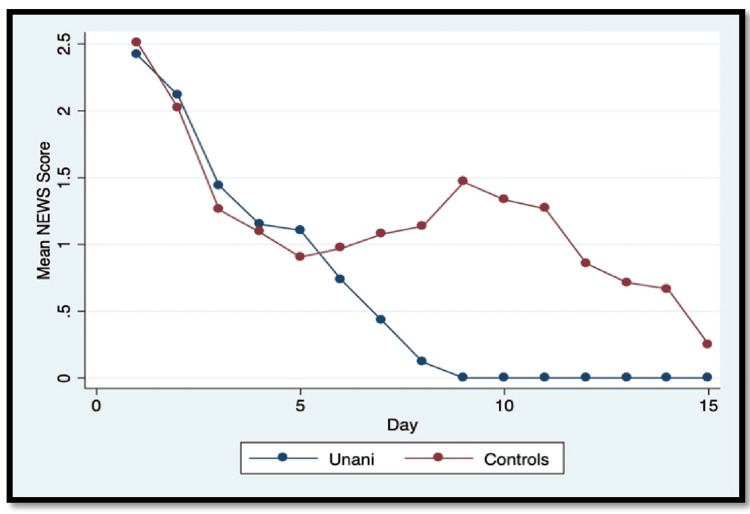
Line chart showing the trend of mean NEWS values among the two arms

NEWS was used to identify patients at risk of early clinical deterioration in hospital settings. NEWS at baseline was comparable between the two arms; in the Unani arm 34 (79.1%), three (6.9%) and six (13.9%) patients, and in the control arm, 37 (78.7%), five (10.6%), and five (10.6%) patients were in low, medium and high risk respectively, and the difference was not statistically significant (p = 0.87). The NEWS considerably decreased at the end of the study in both arms, with a mean difference being 1.5 in the intervention arm (p-value < 0.01) and 1.3 in the control arm (p-value < 0.01) (Figure 3). As seen in Figure 3, the average NEWS score reduced by day 9 in the Unani group, while it was elevated in the control group till day 14.

Duration of supplemental oxygen (in days)

During hospitalization, 12 patients in the Unani arm (27.9%) and eight patients in the control arm (17%) required supplemental oxygen therapy. The mean duration of oxygen requirement was comparatively less in the Unani arm (mean±SD = 2.3±1.5), when compared to the control arm (mean ± SD = 6.0±3.6), the difference between the two arms was statistically significant (p = 0.006).

Mean change in quality-of-life scores on WHO

The impact of the disease on the patient’s quality of life (QoL) was also assessed using WHO Quality of Life-BREF (WHOQoL-Bref), among the four domains: physical health, psychological health, social relationships, and environment. Mean scores of each domain of WHOQoL-Bref were recorded at baseline and discharge; there was no statistical difference noted between the Unani add-on arm and Control arm in either of the domain scores viz. on physical, psychological, social, and environmental (Table 3).

**Table 3:** Assessment of quality of life using WHOQoL-Bref questionnaire Data expressed in mean (SD); p-values were determined using the Chi-square test or Fisher’s exact test for categorical parameters and student t-test for continuous parameters.

	Study arm (Unani add-on) (N = 43)	Control arm (Standard therapy) (N = 47)	Diff. (95%C.I.)	p-value
Physical domain				
Baseline	13.3±0.4	13.1±0.4	-1.8 (-2.3, -1.3)	0.762
Endline	15.1±1.7	14.7±2.5	0.43 (-.49, 1.3)	0.352
p-value	<0.01	<0.01		
Psychological domain				
Baseline	13.1±0.3	13.2±0.4	0.43 (-0.48, 1.4)	0.754
Endline	14.2±1.8	14.2±2.8	0.03 (-0.9, 1.03)	0.950
p-value	<0.01	0.0002		
Social relationships domain				
Baseline	15.7±2.8	15.3±2.8	0.4 (-0.8, 1.5)	0.525
End line	15.9±2.6	15.7±2.9	0.19 (-0.96, 1.4)	0.741
p-value	0.1091	0.0420		
Environmental domain				
Baseline	14.3±2.5	14.6±2.3	-0.14 (-1.2, 0.9)	0.784
End line	14.6±2.3	14.9±2.6	-0.4 (-1.4, 0.7)	0.487
p-value	0.0032	0.0037		

Safety evaluation

In both arms, clinical adverse events (AEs) were recorded every day in a daily assessment record sheet. In three patients of the Unani add-on arm and five patients of the control arm, mild episodes of AEs of anorexia, nausea, abdominal pain, malaise, headache, and heartburn, were observed for a single day, which subsided within a day, and none of them required termination of the treatment. The details are given in Table 2. AEs were notified to the trial site's independent ethics committee (IEC) within the reporting timelines. No serious adverse events (SAEs) or deaths were reported throughout the study. The adverse events were reported less frequently from the patients receiving Unani add-on drugs (9.3%) as compared to the control arm (17%), and this difference was statistically non-significant (p = 0.36).

## Discussion

This study was designed to assess the effectiveness and safety of Unani add-on therapy as compared to the standard treatment in COVID-19 management compared to standard treatment alone. To the best of our knowledge, it is the first study conducted during the second wave of COVID-19 in India evaluating the role of Unani formulations*Tiryaq-e-Arba* and Unani *Joshanda *as an add-on to standard treatment in mild to moderate patients with COVID-19. A total of 90 patients completed the study with male preponderance. The demographic and baseline characteristics were comparable in both arms. The mean age of Unani add-on and control arm was found to be 39.7 and 39.5 years, respectively, and was statistically non-significant (p = 0.94).

The primary outcome of the study was to compare the time taken for the resolution of symptoms of COVID-19 in both arms. The finding showed that the majority of patients who received add-on Unani treatment showed significantly early resolution of symptoms as compared to the control arm (p = 0.02). This early resolution of symptoms in the Unani add-on arm could be attributed to the antiviral, anti-inflammatory, antioxidant, and immunomodulatory activity of the Unani drugs which were used. Among the four components in *Tiryaq-e-Arba*, the aqueous extract of *A. longa* in mice also induce similar immunostimulant activity by increasing the hemagglutinating antibody titer [10]. Yerou et al. showed a positive immunomodulatory response in rats treated with essential oils of *Lauris nobilis* by increasing the white blood cells [14]. *C. Myrrh* exhibits cell-mediated immunity, which is manifested by elevated lymphocytes [13]. Herbal medicinal products having *Gentiana Lutea* root as one of the components show a broad-spectrum antiviral activity in vitro against viruses commonly known to cause respiratory infections. It also exhibits anti-atherosclerotic property due to one of its components, isovitexin [27]. *Myrrh *mediates anti-inflammatory activity by inducing haem oxygenase activity and inhibitory activity against 5-lipoxygenase and helps suppress the production of leukotrienes, which are another class of inflammatory cytokines [12].

The patients were discharged from the hospital as per the protocol issued by the Ministry of Health and Family Welfare. The total number of days a patient spent in the hospital during treatment was recorded and compared with the control arm to know if there was a reduction in the duration of hospital stay. We found that patients who received combination therapy (add-on Unani treatment along with standard treatment) in the intervention arm got early discharge and spent comparatively lesser days in the hospital in contrast to the control arm. Decreased length of stay has been associated with decreased risks of iatrogenic infections, fewer side effects of medication, and reduced unnecessary burden of medical cost on patients, and also helps reduce the patient load on the already overwhelmed health care system, as was observed during the second wave of the pandemic in India [28].

When assessing the need for supplemental oxygen, though a greater number of patients (27.9%) required oxygen supplementation in the Unani arm as compared to the control arm (17.1%), the mean duration of oxygen was significantly less (2.3 days) compared to the control arm (six days {p = 0.006}). This further indicates an early resolution of disease among the patients receiving Unani add-on therapy in the intervention arm.

In addition, though three patients in the control arm required transfer to an ICU during their stay in the hospital, two were in the hospital ICU, and one patient was transferred to another tertiary hospital. None from the Unani arm had to be transferred to an ICU. No deaths were reported in either of the study arms. These results show that add-on Unani therapy could significantly reduce the rate of conversion to severe cases.

The impact of the disease on patient’s quality of life (QoL) was assessed using WHOQOL-Bref. Among the four domains, there was no significant difference noted between the two arms after recovery. However, it was seen that the scores of each domain increased significantly after the treatment in both arms. The social relationship domain score increased marginally and showed statistical non-significance which could be attributed to the social isolation faced by COVID-19 positive patients. The temperament assessment was done in each subject before and after the treatment. It was observed that the *mizaj *remained unchanged even after the treatment in both arms. We found that *Damwi mizaj *(sanguine temperament) was seen predominantly in all patients irrespective of the treatment given. Sanguine is considered a combination of two qualities; hot and moist and according to the Unani concept, infectious disease developed due to abnormal dominance of hot (*Hararat*), moist (*Ratoobat*), and microbes (*Ajsam-e-Khabisa)* [29]. The finding suggested that people of sanguine temperament was associated with increased susceptibility to SARS-CoV-2 infection. These results are not given in the table.

Safety evaluation

The clinical adverse effects were noted in both arms during treatment which were managed conservatively. In both arms, a few patients complained of mild episodes of anorexia, nausea, abdominal pain, malaise, headache, and heartburn, which resolved without withdrawal of treatment (Table 2).

All the clinical studies from AYUSH were reported having safe and effective in the management of COVID-19. The addition Siddha formulations with the standard treatment exhibit early clinical recovery without further progression in mild to moderate COVID-19 cases [30]. A similar study with Unani formulations having similar decoction (*Joshanda*) as an add-on with standard treatment showed accelerated recovery for COVID-19 patients compared to the standard Group [28].

Hence, it can be concluded that the Unani add-on treatment led to a faster resolution of the symptoms and was both safe, and well tolerated. In addition, amongst patients requiring oxygen therapy, it was observed that patients receiving the add-on Unani treatment required relatively less time on supplemental oxygen as opposed to the control arm. Amidst the pandemic when the health systems were struggling with shortage of beds and limited supply of oxygen, resulting in many deaths, the Unani drug as an add-on therapy to standard management was important. These drugs have a potential to curtail the duration of hospitalization and can reduce the burden on the health system during the surge of cases by curtailing the hospital stay of COVID-19 patients.

Strength and limitations

The strength of the study was that it is the first clinical study that evaluated the Unani formulation effectiveness and safety in mild-moderate hospital admitted patients and used clinically relevant outcomes (like time to the resolution of symptoms, and duration of hospital stay) for assessing the efficacy of the formulation.

Our study has some limitations: the open-label study design did not allow the blinding of the clinician. The study had a relatively short duration of follow-up and did not assess the long-term functional outcome/long-term quality of life/progression to “long COVID”. In addition, the study enrolled 90 patients rather than the calculated sample size of 96 due to an abrupt reduction in hospital admissions with the decline of the COVID wave, despite this smaller sample size, the results were statistically significant.

## Conclusions

Unani medicine combined with conventional therapy could be effective and safe in the treatment of mild to moderate COVID-19. Combination therapy can improve the recovery time and clinical cure rate and reduce the rate of conversion to severe cases. However, more high-quality trials are needed in the future to evaluate the efficacy of Unani medicines alone and in combination with conventional therapy in the treatment of mild to moderate COVID-19 patients and could be further tried in post COVID/long COVID cases.

## Appendices

**Table 4:** Composition of polyherbal Unani formulation Tiryaq e-Arba [6,7,10,12,14,16,17,27]

S. No	Botanical Name	Unani Name	Family	Part Used	Quantity	Pharmacological activity
1.	Gentiana lutea L.	Juntiyana	Gentianaceae	roots	1 part	Antiviral Anti-atherosclerotic effects
2.	Aristolochia longa L.	Zarawand Taweel	Aristolochiaceae	roots	1 part	Antioxidant
3.	Commiphora myrrha (Nees) Engl.	Murr makki	Burseraceae	Oleogum resin	1 part	Antiviral, antimicrobial, antioxidant immunomodulator
4.	Laurus nobilis L.	Habb ul Ghar	Lauraceae	berries	1 part	Antiviral, Anti-inflammatory Immunomodulator

**Table 5:** Composition of polyherbal Unani Joshanda 1. Chen J, Tsim KWK: A Review of Edible Jujube, the Ziziphus jujuba Fruit: A Heath Food Supplement for Anemia Prevalence. Front Pharmacol. 2020, 26:593655. 10.3389/fphar.2020.593655 2.  Khanna K, Kohli SK, Kaur R, et al.: Herbal immune-boosters: Substantial warriors of pandemic Covid-19 battle. Phytomedicine. 2021, 85:153361. 10.1016/j.phymed.2020 3. Hamauzu Y, Yasui H, Inno T, Kume C, Omanyuda M.: Phenolic profile, antioxidant property, and anti-influenza viral activity of Chinese quince (Pseudocydonia sinensis Schneid.), quince (Cydonia oblonga Mill.), and apple (Malus domestica Mill.) fruits. J Agri Food Chem. 2005, 53:928-34. 10.1021/jf0494635

S. No	Botanical Name	Unani Name	Family	Part Used	Quantity	Pharmacological activity
1.	Zizyphus jujube	Unnab	Rhamnaceae	Fruits	5 in number	Antiviral, Anti influenza, antiasthamatic, anti -inflammatory, immunomodulator, Antioxidant
2.	Cordia myxa	Sapistan	Boraginaceae	Fruits	9 in number	Antioxidant, immunomodulator
3.	Cydonia oblonga	Behidana	Rosaceae	Seeds	3 gm	Antiviral,antiallergic, antioxidant, antimicrobial, Anti influenza
